# Supplementary material for: Homomeric GluA2(R) AMPA receptors can conduct when desensitized
Source: Nat Commun. 2019 Sep 20;10:4312. doi: 10.1038/s41467-019-12280-9 (PMC6754398; doi:10.1038/s41467-019-12280-9)
Supplement: Supplementary file 1 — Supplementary Information [file 41467_2019_12280_MOESM1_ESM.pdf]

Supplementary Information

Homomeric GluA2(R) AMPA receptors can conduct when desensitized

Coombs *et al.*

This file includes:

Supplementary Discussion.

Supplementary Fig. 1. Current-variance relationships of desensitization for different AMPAR constructs and voltages.

Supplementary Fig. 2. Current-variance relationships of deactivation for edited AMPARs with γ-2.

Supplementary Fig. 3. GluA2(R)/γ-2 receptors at both peak and steady-state mediate negligible Cl^- flux.

Supplementary Fig. 4. Predicted minimum separations of sulfur atoms for mutant cysteines modeled into quisqualate-bound GluA2/γ-2 structures.

Supplementary Fig. 5. Globally averaged currents showing the effects of cross-linking for GluA2 G724C and S729C mutants.

Supplementary Fig. 6. Electron densities of ligands and disulfide bonds for $S729C_{NBQX}$ and $S729C_{ZK}$.

Supplementary Fig. 7. G724C cross-linking disrupts the relaxed dimer structure of LBD.

Supplementary Table 1. List of oligonucleotide sequences.

Supplementary Table 2. Data collection and refinement statistics.

Supplementary Table 3. Details of statistical analyses.

Supplementary References.

Supplementary Discussion

To construct a scheme capable of explaining our kinetic and noise data, we modified the model of Robert and Howe[1]. As with all kinetic schemes, more elaborate versions have the potential to match or improve the quality of fits to the data. However, we aimed to keep modifications to a minimum by including only one additional state (O1), that we previously identified for TARPed receptors[2]. The only transitions added were those linking open states. These were necessary to provide satisfactory fitting of our deactivation noise. The key modification of our model was to allow desensitized states to conduct. We have assumed that these desensitized states have occupancy-dependent conductance (as for the open states).

Could our data be explained by schemes that do not require conducting desensitized channels? AMPARs with or without TARPs are known to exhibit transitions between 'high' and 'low' open probability modes[3,4]. One possibility that we considered is that the atypical behavior of homomeric Q/R-edited AMPARs might arise from 'modal gating', if receptors in these different modes displayed markedly different desensitization properties. Of note, the previous study of Zhang et al.[3] demonstrated that high open probability mode AMPAR gating can yield large relative steady-state currents. However, there is no suggestion that modal behavior could give rise to channels with both a high open probability *and* a low single-channel conductance.

Our evidence strongly suggests that most of the steady-state current of homomeric GluA2 arises from unusually low conductance channel openings of high open probability. While a model in which such openings arose from non-desensitized states could be envisaged, this would require that the receptor rarely resides in the 'classical' desensitized state. Although such a scheme could account for our kinetic and noise data, it would be inconsistent with our observation that highly-desensitizing mutant receptors (S754D and S729C; Fig. 4 and Fig. 6), still conduct current.

Finally, it is important to note that while the introduction of conducting desensitized states into the Robert and Howe model can account for our data, we cannot exclude other kinetic schemes containing such states. For example, our model (and that of Robert and Howe) considers non-desensitized receptors to reside in either gated (O1-O4) or non-gated (R1-R4) states – desensitized receptors might do likewise, i.e. reside in gated (D1*-D4*) or non-gated (D1-D4) states. An alteration in the balance between these gated and non-gated desensitized channels might account for the different steady-state currents of GluA2(R) expressed with different auxiliary subunits. Such a model would necessitate the incorporation of an additional 7 states and at least 2 additional rate constants. Based on our data, we have no reason to favor this more complicated model.

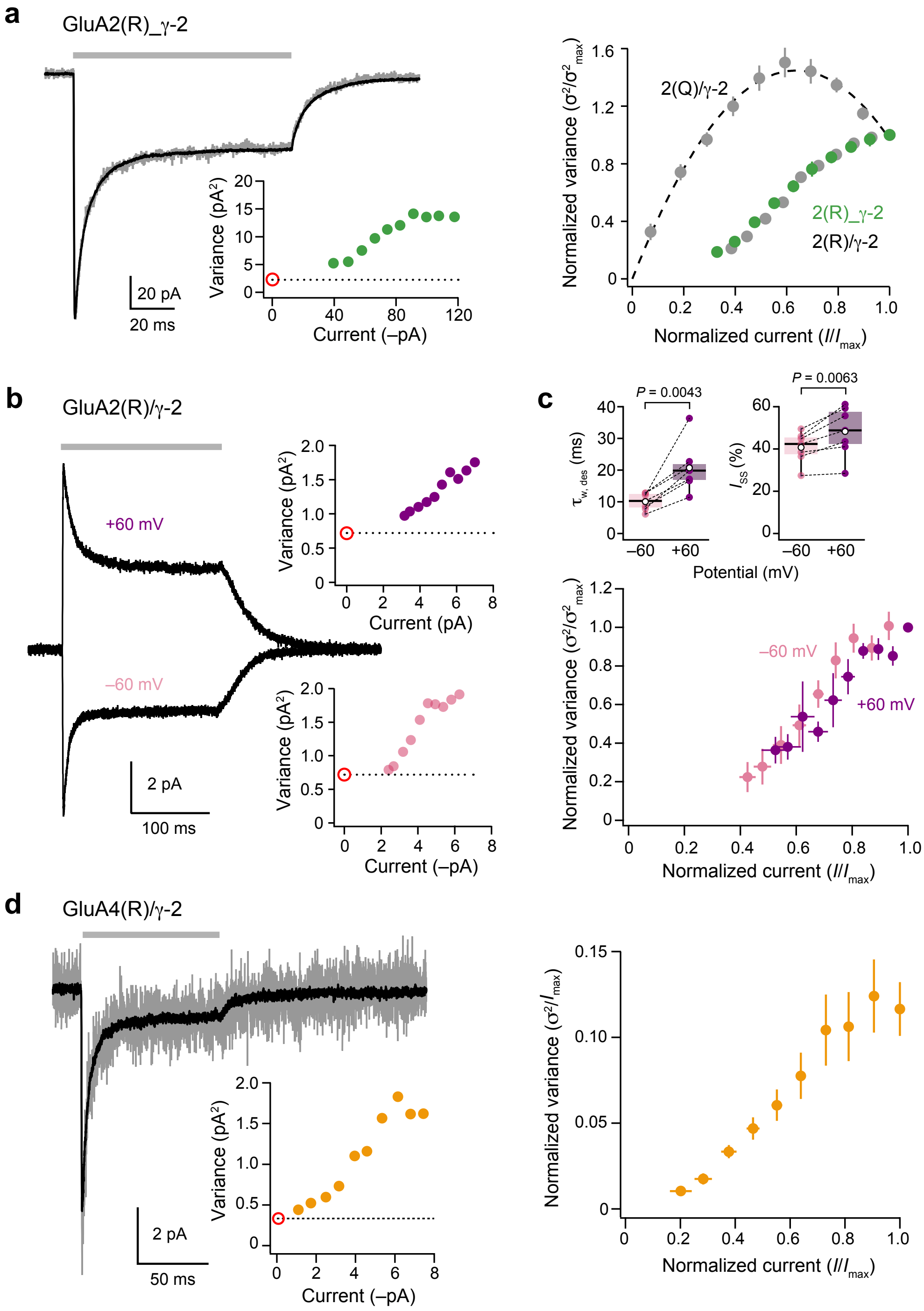

Supplementary Figure 1. Current-variance relationships of desensitization for different AMPAR constructs and voltages. **a**) Representative current (gray; average in black) from an outside-out patch taken from a tandem GluA2(R)_γ-2-expressing HEK293 cell, evoked by 100 ms application of 10 mM glutamate at –60 mV (gray bar). Inset: current-variance relationship for the same patch (dotted line indicates background variance and open red circle indicates expected origin). Note that the current-variance relationship cannot be fitted with a parabola passing through the origin. Right panel shows doubly normalized and averaged current-variance relationships for desensitization of GluA2(R)_γ-2 (green circles; $n = 8$). Also shown, for comparison, are corresponding relationships for GluA2(Q)/γ-2 and GluA2(R)/γ-2 (gray circles, from Fig. 1). Error bars denote sems. **b**) Averaged GluA2(R)/γ-2 currents evoked by 200 ms applications of 10 mM glutamate (gray bar) at +60 and –60 mV. Insets: current-variance relationships for the same responses (displayed as in a). Note that in neither case can the current-variance relationship be fitted with a parabola passing through the origin. **c**) Upper panels show pooled data for kinetic properties ($n = 7$ for both). Box-and-whisker plots as in Fig. 1c, but with dashed lines indicating paired measurements. Indicated P values are from paired t-tests. Lower panel shows doubly normalized and averaged current-variance relationships for desensitization of GluA2(R)/γ-2 at +60 and –60 mV ($n = 5$ and 6, respectively). Error bars denote sems. **d**) Representative GluA4(R)/γ-2 current (gray, average in black) evoked by 100 ms application of 10 mM glutamate at –60 mV. Inset: current-variance relationship for the same patch (displayed as in a). Note that the current-variance relationship cannot be fitted with a parabolic relationship passing through the origin. Right panel shows normalized and averaged current-variance relationships for desensitization of GluA4(R)/γ-2 ($n = 6$). Error bars denote sems. Source data are provided as Source Data file.

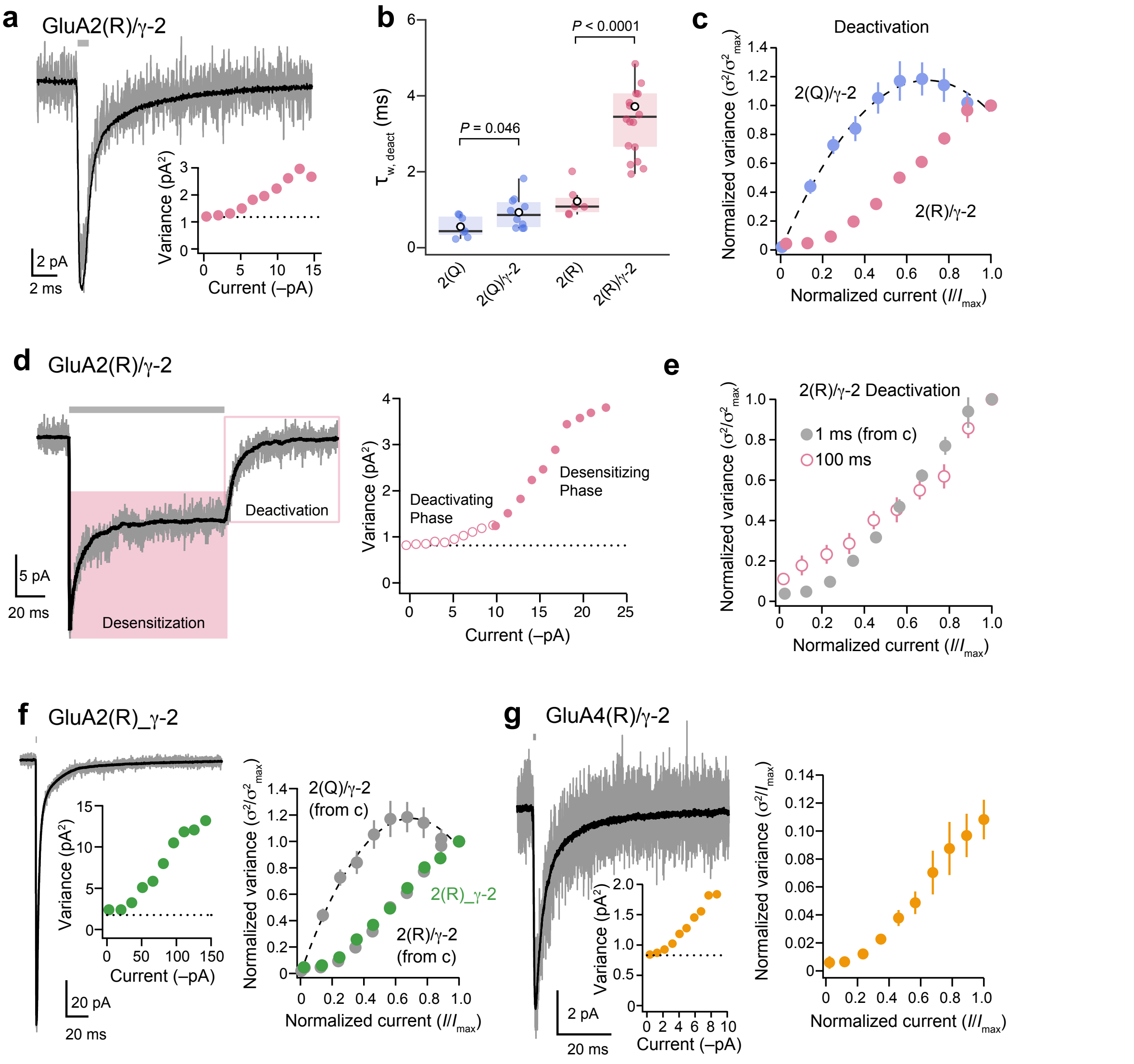

Supplementary Figure 2. Current-variance relationships of deactivation for edited AMPARs with γ-2. a) Representative GluA2(R)/γ-2 current (gray, average in black) evoked by 1 ms application of 10 mM glutamate at –60 mV (gray bar) to an outside-out patch from a transfected HEK293 cell. Inset: current-variance relationship for the same patch (dotted line indicates background variance). Note that the data can not be fitted with a parabola. **b**) Pooled deactivation kinetics for GluA2(Q) and (R) with and without γ-2 (for Q *n* = 10 and 7; for R *n* = 18 and 6, respectively). Box-and-whisker plot as in Fig. 1c. Two-way ANOVA indicated an effect of Q/R editing ($F_{1,37}$ = 34.70, P < 0.0001), an effect of γ-2 ($F_{1,37}$ = 17.47, P = 0.00017) and a significant interaction ($F_{1,37}$ = 20.64, P < 0.0001). The mean difference in $\tau_{w,\ deact}$ between GluA2 with- and without γ-2 was 0.38 ms (95% confidence interval, 0.086 to 0.72) for Q, and 2.5 ms (95% confidence interval, 1.83; 3.41) for R. Indicated *P* values are from unpaired Welch two-sample *t*-tests. **c**) Doubly normalized and averaged current variance relationships of deactivation for GluA2(Q)/γ-2 (*n* = 7) and GluA2(R)/γ-2 (*n* = 18). Error bars denote sems. **d**) A representative GluA2(R)/γ-2 current evoked by a 100 ms application of 10 mM glutamate at –60 mV. NSFA was performed on both the desensitizing phase (pink shaded region; as performed in Fig. 1), and on the deactivating phase following glutamate removal (open pink box). Right panel shows current-variance relationship for both phases of the current. **e**) Doubly normalized current-variance relationships for deactivation of GluA2(R)/γ-2 (1 ms, duplicated from c; 100 ms, *n* = 11). Note the similarity in shape of the relationships. Error bars indicate sems. **f**) Deactivation current and current-variance relationship for the tandem construct GluA2(R)_γ-2. Representative trace in gray and average in black. The right hand panel shows pooled doubly normalized current and variance (*n* = 5) compared with separately expressed subunits (from c). Error bars indicate sems. **g**) As for f, but for GluA4(R)/γ-2 deactivation (*n* = 5). Error bars indicate sems. Source data are provided as a Source Data file.

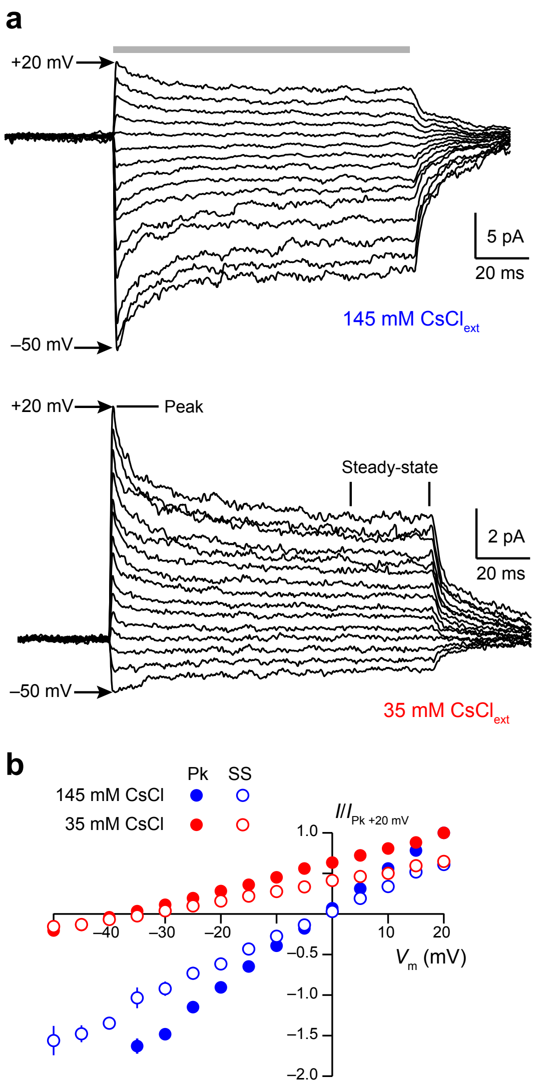

Supplementary Figure 3. GluA2(R)/γ-2 receptors at both peak and steady-state mediate negligible Cl⁻ flux. **a**) Representative GluA2(R)/γ-2 currents evoked by 100 ms applications of 10 mM glutamate (gray bar) to an outside-out patch from a transfected HEK293 cell (at potentials between –50 and +20 mV; Δ5 mV). Currents were recorded in external solution containing either 145 mM CsCl, or 35 mM CsCl. **b**) Mean current-voltage relationships (normalized to the peak current at +20 mV) for the high CsCl solution (blue) and the low CsCl solution (red) (*n* = 7). Error bars denote sems but are smaller than the symbols in most cases. Note the similar shift in reversal potential of both the peak (Pk) and steady-state (SS) components. Analysis of individual current-voltage relationships showed that the reversal potentials (mean ± sem) in 'high' Cs were –1.7 ± 0.6 mV for peak and –1.1 ± 0.5 mV for steady-state. The corresponding values in 'low' Cs were –37.6 ± 1.4 mV and –34.8 ± 1.7 mV.

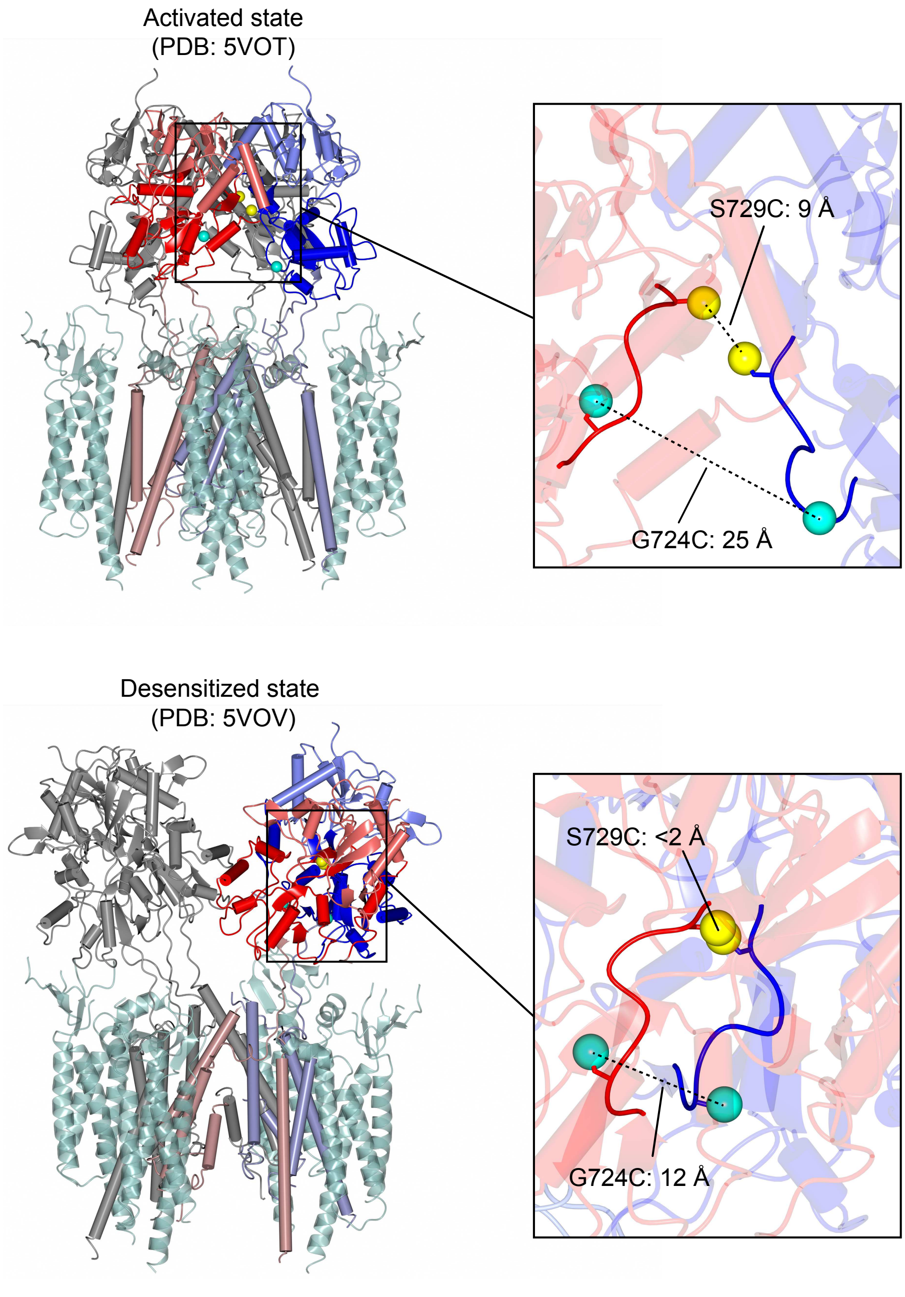

Supplementary Figure 4. Predicted minimum separations of sulfur atoms for mutant cysteines modeled into quisqualate-bound GluA2/γ-2 structures. Cysteine substitutions at Ser729 and Gly724 were modeled into cryo-EM structures of GluA2(R)/γ-2 in the activated state (upper) and desensitized-like state (lower) (from Ref. 5). Subunits 'A' and 'D' are shown in red and blue, respectively, with subunits 'B' and 'C' (gray) and all four γ-2 subunits (sea green, transparent). Expanded sections highlight the sulfur atoms of the modeled cysteines at positions 724 (cyan) and 729 (yellow), with the calculated separations. The modeling predicts that a disulfide bond (2 Å) can only be accommodated by desensitized GluA2(R) S729C/γ-2.

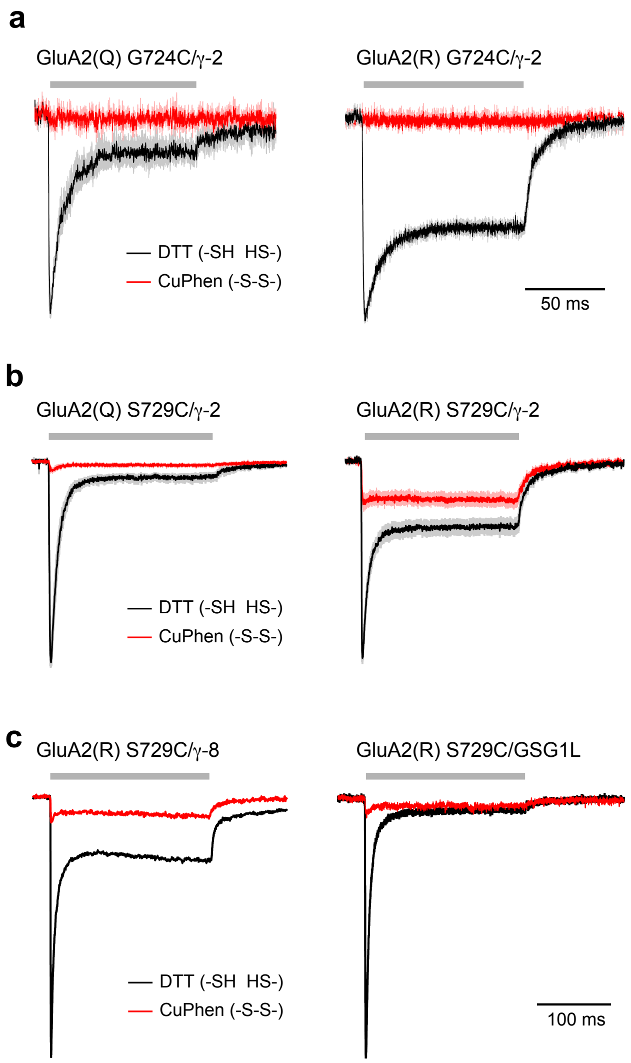

Supplementary Figure 5. Globally averaged currents showing the effects of cross-linking for GluA2 G724C and S729C mutants. **a**) Global averaged currents at –60 mV activated by 10 mM glutamate (100 ms; gray bar) from unedited and edited GluA2 G724C/γ-2 (n = 6 and 5). Note that, GluA2(R) G724C/γ-2 is fully inhibited following cross-linking by 10 μM CuPhen. **b**) Same as a, but for GluA2 S729C/γ-2 (n = 10 and 9). Note that, unlike G724C, GluA2(R) S729C is not fully inhibited following cross-linking. In each case lighter shading indicates sem. **c**) Global averaged currents showing that cross-linking of GluA2(R) S729C is unable to fully inhibit steady-state currents when the receptors are co-expressed with γ-8 or GSG1L (n = 4 for both). Note that the steady-state current remaining after cross-linking (normalized to the peak current in control; $I_{SS\text{-}CuPhen}/I_{Pk\text{-}DTT}$) was 0.19, 0.07, and 0.03, for GluA2(R) S729C expressed with γ-2, γ-8 or GSG1L.

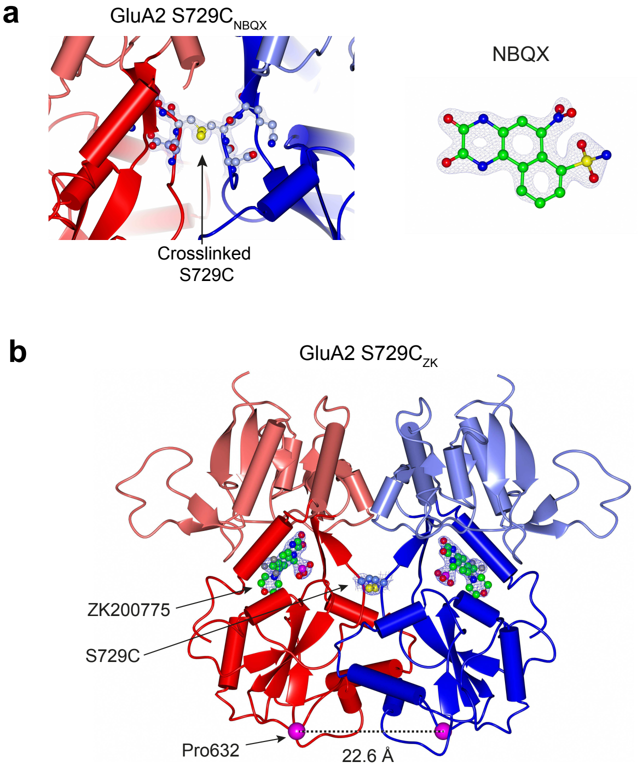

Supplementary Figure 6. Electron densities of ligands and disulfide bonds for S729C$_{NBQX}$ and S729C$_{ZK}$. **a**) 2F$_o$-F$_c$ electron densities around residues 728-730, including the S729C disulfide bond (contoured at 2.0 σ) and around NBQX from S729C$_{NBQX}$ subunit 'A' (contoured at 2.5 σ). **b**) Structure of GluA2 S729C$_{ZK}$, which is highly similar to that of S729C$_{NBQX}$ (Fig. 7) (RMSD = 0.65 Å). Monomers are colored red and blue. Arrows indicate the ligand and the S729C disulfide bond (with their 2F$_o$-F$_c$ electron density contoured at 2.0 σ), and the Cα atoms of Pro632 residues (magenta spheres).

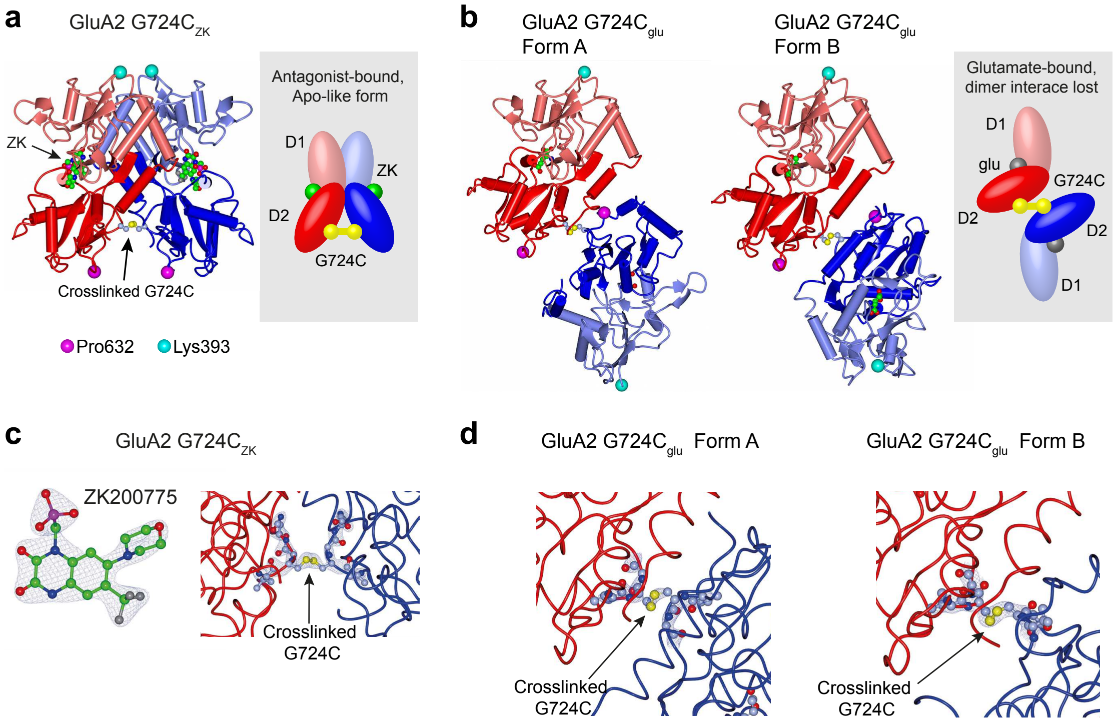

Supplementary Figure 7. G724C cross-linking disrupts the relaxed dimer structure of LBD. **a**) Crystal structure of the dimeric GluA2 G724C ligand binding core in the presence of ZK200775 (ZK). There were eight monomers in the unit cell, only Monomer 'A' (red) and Monomer 'H' (blue) are displayed. Cα atoms of Lys393 positioned at the ATD-LBD linker (cyan spheres) and proline 632 at the M3-D2 linker (magenta spheres) emerge on the same sides of the dimer consistent with the expected LBD orientation within full length structures. Cartoon illustrates the position of the disulfide bond (yellow) between D2 lobes of the two monomers. **b**) Two forms of the glutamate-bound GluA2 G724C ligand binding core. D1 lobes of red monomers are displayed in the same orientation as in a. Unlike the antagonist bound form, the monomers undergo a large relative rotation compared with wild-type forms. While pairs of Pro632 residues are adjacent, Lys393 residues are positioned on opposite sides of the complex, which does not form a 'relaxed dimer' (see cartoon). **c**) $2F_o$-F_c electron density around ZK200775 (subunit 'A', from a), (contoured at 2.0 σ) and the G724C disulfide bond (subunit 'A' and 'H'), (contoured at 2.5 σ). **d**) $2F_o$-F_c electron density around the G724C disulfide bond (contoured at 2.0 σ) for both forms of G724C$_{glu}$.

Supplementary Table 1

List of oligonucleotide sequences.

Point mutations	Direction	5′–3′
S754D	F	GTATTGAAACTCGATGAGCAAGGC
	R	GCCTTGCTCATCGAGTTTCAATAC
G724C	F	CATGAAAGTGTGCGGAAACTTGG
	R	CCAAGTTTCCGCACACTTTCATG
S729C	F	GAAACTTGGATTGCAAAGGCTAC
	R	GTAGCCTTTGCAATCCAAGTTTC

Supplementary Table 2

Data collection and refinement statistics.

	G724C	G724C	G724C	S729C	S729C
Additives	ZK200775	Glutamate (Form A)	Glutamate (Form B)	NBQX	ZK200775
PDB code	6FQJ	6FQG	6FQI	6FQH	6FQK
**Data collection**					
Beamline	DLS I24	ESRF ID 30B	DLS I04	DLS I04	DLS I04
Space group	P 21 21 21	P 1 21 1	P 41	P 61	P 61
Cell dimensions a, b, c (Å)	86.01, 144.29, 199.18	50.465, 87.655, 68.280	50.65, 50.65, 256.29	108.12, 108.12, 99.13	106.70, 106.70, 100.39
Cell angles α, β, γ (Å)	90, 90, 90	90, 110.223, 90	90, 90, 90	90, 90, 120	90, 90, 120
Wavelength (Å)	0.9686	0.97623	0.9795	0.9795	0.9795
Resolution (Å)	81.96-2.5 (2.59-2.50)	51.73-2.34 (2.43-2.34)	49.69-2.91 (3.01-2.91)	28.8-1.76 (1.82-1.76)	92.41-1.98 (2.05-1.98)
R_{meas}	0.121 (0.499)	0.098 (0.769)	0.121 (0.887)	0.041 (1.331)	0.06 (1.323)
R_{pim}	0.086 (0.353)	0.052 (0.408)	0.068 (0.493)	0.016 (0.574)	0.024 (0.509)
Half-set correlation $CC_{1/2}$ (%)	0.985 (0.736)	0.997 (0.765)	0.995 (0.613)	1 (0.537)	0.997 (0.602)
$I/\sigma I$	6.8 (2.2)	9.8 (1.8)	10.7 (1.7)	23.1 (1.4)	17.2 (1.5)
Completeness (%)	99.9 (100.0)	96.9 (93.2)	99.6 (99.9)	100.0 (100.0)	100.0 (99.7)
Multiplicity	1.9 (1.9)	3.4 (3.4)	3.1 (3.1)	6.7 (5.3)	6.5 (6.6)
**Refinement**					
Resolution (Å)	81.96-2.50 (2.59-2.50)	51.73-2.34 (2.43-2.34)	49.69-2.91 (3.01-2.91)	28.8-1.76 (1.82-1.76)	53.35-1.98 (2.05-1.98)
No. of reflections	86340	22793	13987	64664	43434
R_{work}/R_{free}	0.199/0.265	0.179/0.233	0.214/0.267	0.189/0.224	0.218/0.256
No. of atoms	17200	4166	4029	4531	4385
Average *B*-factors – Protein	49.04	50.43	71.43	50.47	53.80
Average *B*-factors – Water	37.49	46.68		48.68	48.78
Average *B*-factors – Ligand	39.78	41.80	39.86	51.53	44.04
Rmsd Bond lengths (Å)	0.013	0.013	0.003	0.011	0.009
Rmsd Bond angles (°)	1.25	1.25	0.70	1.04	1.13
Ramachandran favored/outliers (%)	97.3/0.2	96.5/0.0	94.8/0.4	97.7/0.2	98.3/0.0

Supplementary Table 3

Details of statistical analyses.

Figure	Test	Description	Statistic	*P*-value	Method
**Fig. 1c** $\tau_{w, des}$	2-way ANOVA	Main effect of Q/R editing	$F_{1,97}$ = 111.34	2.20 e-16	Welch heteroscedastic *F*-test
		Main effect of auxiliary subunit type	$F_{3,97}$ = 32.30	1.45 e-14	Welch heteroscedastic *F*-test
		Interaction	$F_{3,97}$ = 2.84	0.041	Welch heteroscedastic *F*-test
	Pairwise tests	GluA2(Q) *vs* GluA2(R)	$t_{15.58}$ = 10.26	2.48 e-08	Welch two-sample (two-sided) t-test
		GluA2(Q)/γ-2 *vs* GluA2(R)/γ-2	$t_{43.01}$ = 2.39	0.021	Welch two-sample (two-sided) *t*-test
		GluA2(Q)/γ-8 *vs* GluA2(R)/γ-8	$t_{15.00}$ = 3.43	0.0037	Welch two-sample (two-sided) *t*-test
		GluA2(Q)/GSG1L *vs* GluA2(R)/GSG1L	$t_{15.99}$ = 3.24	0.0052	Welch two-sample (two-sided) *t*-test
**Fig. 1d** I_{SS}	2-way ANOVA	Main effect of Q/R editing	$F_{1,97}$ = 129.98	1.31 e-19	Welch heteroscedastic *F*-test
		Main effect auxiliary subunit type	$F_{3,97}$ = 58.30	1.24 e-21	Welch heteroscedastic *F*-test
		Interaction	$F_{3,97}$ = 58.67	1.02 e-21	Welch heteroscedastic *F*-test
	Pairwise tests	GluA2(Q) *vs* GluA2(R)	$t_{8.95}$ = 7.70	3.07 e-05	Welch two-sample (two-sided) *t*-test
		GluA2(Q)/γ-2 *vs* GluA2(R)/γ-2	$t_{33.57}$ = 14.32	7.35 e-16	Welch two-sample (two-sided) *t*-test
		GluA2(Q)/γ-8 *vs* GluA2(R)/γ-8	$t_{9.70}$ = 7.76	1.82 e-05	Welch two-sample (two-sided) *t*-test
		GluA2(Q)/GSG1L *vs* GluA2(R)/GSG1L	$t_{8.88}$ = 1.42	0.19	Welch two-sample (two-sided) *t*-test
**Fig. 1f** γ	Pairwise tests	GluA2(Q) *vs* GluA2(Q)/γ-2	$t_{26.93}$ = 6.98	5.06 e-7	Welch two-sample (two-sided) *t*-test
		GluA2(Q) *vs* GluA2(Q)/γ-8	$t_{6.86}$ = 3.67	0.016	Welch two-sample (two-sided) *t*-test
		GluA2(Q) *vs* GluA2(Q)/GSG1L	$t_{15.68}$ = 2.98	0.016	Welch two-sample (two-sided) *t*-test
**Fig. 3a** γ	Pairwise test	GluA2(Q)/γ-2 Activation *vs* Deact.	t_{4} = 0.51	0.64	Paired *t*-test
**Fig. 4c** $\tau_{w, des}$	2-way ANOVA	Main effect of Q/R editing	$F_{1, 17}$ = 10.56	0.0047	Welch heteroscedastic *F*-test
		Main effect of S754D mutation	$F_{1, 17}$ = 43.19	4.75 e-6	Welch heteroscedastic *F*-test
		Interaction	$F_{1, 17}$ = *2.63*	0.12	Welch heteroscedastic *F*-test
	Pairwise tests	GluA2(Q)/γ-2 *vs* GluA2(Q)/γ-2 S754D	$t_{5.00}$ = 5.85	0.0021	Welch two-sample (two-sided) *t*-test
		GluA2(R)/γ-2 *vs* GluA2(R)/γ-2 S754D	$t_{4.00}$ = 4.64	0.0097	Welch two-sample (two-sided) *t*-test
**Fig. 4d** $\tau_{w, recov}$	2-way ANOVA	Main effect of Q/R editing	$F_{1, 17}$ = 0.13	0.72	Welch heteroscedastic *F*-test
		Main effect of S754D mutation	$F_{1, 17}$ = 31.67	3.01 e-5	Welch heteroscedastic *F*-test
		Interaction	$F_{1, 17}$ = 1.65	0.22	Welch heteroscedastic *F*-test
	Pairwise tests	GluA2(Q)/γ-2 *vs* GluA2(Q)/γ-2 S754D	$t_{6.24}$ = 4.68	0.0030	Welch two-sample (two-sided) *t*-test
		GluA2(R)/γ-2 *vs* GluA2(R)/γ-2 S754D	$t_{5.09}$ = 4.42	0.0066	Welch two-sample (two-sided) *t*-test
**Fig. 4e** I_{SS}	2-way ANOVA	Main effect of Q/R editing	$F_{1, 17}$ = 65.37	3.16 e-7	Welch heteroscedastic *F*-test
		Main effect of S754D mutation	$F_{1, 17}$ = 28.72	5.56 e-5	Welch heteroscedastic *F*-test
		Interaction	$F_{1, 17}$ = 14.93	0.0012	Welch heteroscedastic *F*-test
	Pairwise tests	GluA2(Q)/γ-2 *vs* GluA2(Q)/γ-2 S754D	$t_{5.03}$ = 4.71	0.0052	Welch two-sample (two-sided) *t*-test
		GluA2(R)/γ-2 *vs* GluA2(R)/γ-2 S754D	$t_{4.23}$ = 5.57	0.0043	Welch two-sample (two-sided) *t*-test
**Fig. S1c** $\tau_{w, des}$	Pairwise test	GluA2(Q) *vs* GluA2(Q)/γ-2	t_{6} = 4.44	0.0043	Paired *t*-test
I_{SS}	Pairwise test	GluA2(Q) *vs* GluA2(Q)/γ-2	t_{6} = 4.11	0.0063	Paired *t*-test
**Fig. S2b** $\tau_{w, deact}$	2-way ANOVA	Main effect of Q/R editing	$F_{1, 37}$ = 34.70	8.82 e-7	Welch heteroscedastic *F*-test
		Main effect of γ-2	$F_{1, 37}$ = 17.47	1.71 e-4	Welch heteroscedastic *F*-test
		Interaction	$F_{1, 37}$ = 20.64	5.74 e-5	Welch heteroscedastic *F*-test
	Pairwise tests	GluA2(Q) *vs* GluA2(Q)/γ-2	$t_{14.97}$ = 2.17	0.046	Welch two-sample (two-sided) *t*-test
		GluA2(R) *vs* GluA2(R)/γ-2	$t_{21.72}$ = 6.06	4.41 e-6	Welch two-sample (two-sided) *t*-test
**Fig. S3** I_{rev} shift	Pairwise test	GluA2(R)/γ-2 peak *vs* steady-state	t_{6} = 1.94	0.10	Paired *t*-test

Supplementary References

1. Robert, A. & Howe, J.R. How AMPA receptor desensitization depends on receptor occupancy. *J Neurosci* **23**, 847-858 (2003).

2. Coombs, I.D., MacLean, D.M., Jayaraman, V., Farrant, M. & Cull-Candy, S.G. Dual effects of TARP γ-2 on glutamate efficacy can account for AMPA receptor autoinactivation. *Cell Rep* **20**, 1123-1135 (2017).

3. Zhang, W., Devi, S.P., Tomita, S. & Howe, J.R. Auxiliary proteins promote modal gating of AMPA- and kainate-type glutamate receptors. *Eur J Neurosci* **39**, 1138-1147 (2014).

4. Prieto, M.L. & Wollmuth, L.P. Gating modes in AMPA receptors. *J Neurosci* **30**, 4449-4459 (2010).

5. Chen, S., et al. Activation and desensitization mechanism of AMPA receptor-TARP complex by cryo-EM. *Cell* **170**, 1234-1246 (2017).
